# Changes in Nutrition, Physical Activity, and Body Weight among Lithuanian Students during and after the COVID-19 Pandemic

**DOI:** 10.3390/nu15184091

**Published:** 2023-09-21

**Authors:** Vilma Kriaučionienė, Monika Grincaitė, Asta Raskilienė, Janina Petkevičienė

**Affiliations:** 1 Health Research Institute, Faculty of Public Health, Lithuanian University of Health Sciences, Tilzes Str. 18, 47181 Kaunas, Lithuania; 2 Department of Preventive Medicine, Faculty of Public Health, Lithuanian University of Health Sciences, Tilzes Str. 18, 47181 Kaunas, Lithuania

**Keywords:** COVID-19 pandemic, post-pandemic period, students, nutrition habits, physical activity, weight changes

## Abstract

The long-term effect of the COVID-19 pandemic on lifestyle changes remains understudied. This cross-sectional study aimed to assess changes in nutrition, physical activity, and body weight among Lithuanian students during the pandemic and their post-pandemic persistence. Data were collected from 1430 first-year students (325 males, 1105 females) through an online self-administered questionnaire. The students indicated an increase in the consumption of snacks, fast food, home-made confectionery, and sweets. A decrease in physical activity during the pandemic was reported by 34.9% of males and 33.2% of females. Lifestyle changes during the pandemic were associated with post-pandemic habits. A higher proportion of females (38.7%) than males (31.1%) indicated an increase in BMI, which was more pronounced among students with overweight. A multivariate logistic regression analysis revealed that reduced physical activity; the increased consumption of red meat, snacks, fast food, and home-made confectionery during the pandemic; and post-pandemic BMI were linked with weight gain. After the pandemic, weight gain persisted in 40.9% of students who continued to follow unhealthy nutrition habits and had reduced physical activity. The study emphasizes that the promotion of a healthy diet and regular physical activity among university students is essential for developing lifelong habits that contribute to a healthy body weight and overall well-being.

## 1. Introduction

First-year students are a dynamic group that experiences many challenges, changes, and opportunities during their transition to higher education. Previous studies have identified unhealthy shifts in their dietary habits and physical activity, linking the changes with weight gain [1,2,3]. In Lithuania, the prevalence of overweight and obesity increased among first-year male students (from 11.3% in 2000 to 24.3% in 2017) and female students (from 5.2 to 9.6%) [3]. The COVID-19 pandemic could potentially have exacerbated these shifts.

The sudden onset of the coronavirus disease (COVID-19) has changed the everyday lives of many people. The countries have taken urgent and strict measures to control the spread of the virus. The Lithuanian Government announced a quarantine on 16 March 2020 [4]. In Lithuania, the quarantine lasted more than a year, until 1 July 2021, with varying levels of restrictions. Most workspaces, schools, universities, sports venues, and shopping centers were closed. Educational institutions started distance learning. Drastic changes in the educational process, social isolation, constant anxiety, and uncertainty began to negatively affected the daily life of students [5]. Several studies showed changes in students’ dietary habits, with decreased breakfast consumption, increased snacking between meals, and increased total energy intake [6,7]. In addition, the students consumed less health-promoting foods like grains, dairy products, nuts, fruits, and vegetables. Conversely, there was an increase in the consumption of confectionery, sweets, and fast food [6,8,9,10,11]. Females tended to consume more healthy foods and fewer unhealthy snacks, fast food, soft drinks, meat and meat products, and fermented cheese than males [12,13,14,15].

Studies conducted in Southern European countries showed increased consumption of legumes, fish, eggs, yoghurt, fruits, and vegetables [7,16,17]. Another study found that students decreased their consumption of processed meat, sausages, and frozen semi-prepared meals [5].

The decreasing level of physical activity among university students has become a global concern in recent years [18,19]. The pandemic further exacerbated this issue, with a decline in physical activity among students. More than 40% of Italian, 59% of Chinese, and 60% of Polish students reported lower physical activity than before the pandemic [16,20,21]. The duration of moderate-intensity physical activity decreased by one-third among Spanish and French students [22,23].

Remote learning resulted in a larger proportion of students spending more time sitting in front of electronic devices (e.g., computers, tablets, smartphones, and television screens) [17,24]. Studies showed longer sitting times compared to pre-quarantine in Spanish, Italian, Saudi Arabian, and Canadian students [16,25,26,27]. The time spent on screens increased more than twice among U.S. students [24], and it tripled among Croatian students [17]. A study demonstrated that increased screen time and reduced physical activity were associated with unhealthy dietary habits [28].

Changes in diet and physical activity during the COVID-19 pandemic have had an impact on the body weight of students, with a substantial proportion experiencing weight gain. The increase in body weight was more pronounced among students with overweight [29].

Despite numerous studies exploring changes in students’ nutrition habits, physical activity, and body weight during the COVID-19 pandemic, a significant gap remains in understanding the pandemic’s long-term effects on the lifestyle and health of young people. Under certain circumstances, temporary alterations in dietary and physical activity habits can become a regular part of students’ daily routines. Reversing lifestyle changes caused by the pandemic and reducing excess body weight can be challenging. Insights into students’ post-COVID-19 lifestyle choices could aid in creating proactive strategies to address adverse lifestyle changes and related health issues. This study aimed to assess changes in nutrition, physical activity, and body weight among Lithuanian students during the pandemic and their persistence post-pandemic.

## 2. Materials and Methods

### 2.1. Study Design and Sample

The online cross-sectional survey was conducted among first-year students of the largest Lithuanian Universities of Applied Sciences—Vilnius University of Applied Sciences, Kaunas University of Applied Sciences, Klaipeda State University of Applied Sciences, and Siauliai State University of Applied Sciences—in the second semester of the 2021–2022 academic year, specifically in April 2022. The data collection process involved sending emails to all students of the selected faculties with information about the survey and invitation to participate. The online survey was open for three weeks. Additional reminders were sent during the first and second weeks to encourage questionnaire completion.

Participation in the study was voluntary and anonymous, ensuring that individuals felt comfortable providing their responses. Out of the initial 1494 respondents, a total of 64 participants (4.5%) did not provide all the required information. Consequently, these individuals were excluded from the analysis, resulting in a final dataset of 1430 students, comprising 325 males and 1105 females.

The study protocol received ethical approval from the Bioethics Centre at the Lithuanian University of Health Sciences (protocols BC-GVM(M)-80, BC-GVM(M)-118, and BEC-GVM(M)-119). Additionally, permission to conduct the study was obtained from the administration of the participating universities, ensuring compliance with institutional regulations and policies.

### 2.2. Measurements

A standardized questionnaire developed for this study was used. Questions about dietary patterns, physical activity level, body weight, and changes in lifestyle habits during and after the COVID-19 pandemic were included. 

A food frequency questionnaire was employed to assess the students’ dietary habits [30]. The respondents were asked to indicate the frequency of consumption of various food items, such as meat, fish, milk and dairy products, cereal products, fresh vegetables, fruits, confectionery, sweets, soft drinks, fast food, unhealthy snacks, etc. Response options included the following categories: ‘several times a day’, ‘daily’, ‘several times a week’, ‘1–4 times a month’, and ‘never’. Based on the reported frequency of food consumption, respondents were categorized into three groups: (1) ‘daily consumption’ (‘several times per day’ or ‘daily’), (2) several times a week, and (3) 1–4 times a month or less frequently (‘1–4 times a month’ or ‘never’). 

Physical activity was evaluated by asking the following question: ‘In your leisure time, how often do you do physical exercise for at least 30 min, which makes you at least mildly short of breath or perspire?’ According to the answers, the respondents were grouped into two groups: physically active at least four times a week and physically active less often than this.

The participants were asked to report their actual weight in kilograms and height in centimeters. Subsequently, body mass index (BMI) was calculated by dividing the weight (in kg) by the square of the height (in meters). BMI values were categorized as follows: underweight (BMI < 18.5 kg/m²), normal weight (BMI 18.5–24.9 kg/m²), overweight (BMI 25–29.9 kg/m²), and obesity (BMI > 30 kg/m²).

Regarding the impact of the COVID-19 quarantine, students were queried about changes in various lifestyle factors, including diet, physical activity, and body weight, compared to the usual routine. The question was: ‘How did the listed lifestyle factors and body weight change during the COVID quarantine compared to your usual lifestyle and body weight?’ Response options included: ‘increased’, ‘remained as usual’, and ‘decreased’. In terms of weight change, respondents were categorized as those who gained weight and others (‘remained as usual’ or ‘decreased’). Additionally, participants were asked about changes after the pandemic: ‘Have the listed lifestyle factors and body weight returned to their usual routine after the COVID-19 quarantine?’ The possible answers were: ‘the changes remained’ and ‘the changes returned to the usual routine’.

### 2.3. Statistical Analysis

Data analysis was conducted using the IBM SPSS Statistics software package, version 29.0 (IBM Corp.: Armonk, NY, USA, released 2022). Categorical variables were presented as percentages and compared using the chi-square test. For multiple comparisons, the z-test with Bonferroni correction was applied. Logistic regression analysis was used to evaluate the associations between the increased consumption of certain foods during the COVID-19 pandemic (dependent variable) and consumption of the same foods after the pandemic. A separate logistic regression analysis adjusted for sex and age was performed for every food item. The logistic regression analysis was also used to assess associations between weight gain (dependent variable) and changes in nutrition and physical activity during the COVID-19 pandemic and BMI after the pandemic. First, a univariate logistic regression for every variable, such as sex, age, increased food consumption and decreased physical activity during the COVID-19 pandemic, and BMI after the pandemic, was performed. Next, multivariate stepwise logistic regression analysis was used by including all potential independent variables in the model and eliminating those that were not statistically significant. A significance level of 0.05 was chosen as the threshold for statistical hypothesis testing.

## 3. Results

The main characteristics of the study population are presented in Table 1. The proportion of female students was higher than male students. Moreover, comparable proportions of students were observed in the age groups of 18–19 years and 20 years or older. Notably, a significant proportion of students were from the two major universities, located in the capital city Vilnius and the second largest city Kaunas, which collectively contribute to a substantial portion of the study population. However, students from various regions of Lithuania were studying at these universities. According to the self-reported weight and height data, the majority of students had normal BMI (Table 1).

A substantial number of students reported significant changes in their eating habits during the quarantine period, as revealed in Table 2. More than one-third of males (33.7%) and 24.1% of females indicated an increase in snacking behavior. The consumption of fast food, home-made confectionery, and sweets increased among over one-fourth of males. Conversely, the rise in the consumption of these food items was comparatively less pronounced among females. Interestingly, although a larger proportion of males reported an increase in the consumption of fresh, boiled, and pickled vegetables, a greater number of females indicated maintaining the same frequency of consumption as before the quarantine. Furthermore, approximately one-fifth of respondents reported an increase in the amount of food ordered for home delivery or takeaway. In terms of beverage consumption, a greater number of students indicated a reduction in the usage of carbonated and sugary drinks compared to those who reported an increase. A decrease in physical activity was reported by more than one-third of the students. A higher proportion of females than males reported an increase in their BMI during the COVID-19 pandemic: 38.7% and 31.1%, respectively. Body weight decreased in 14.5% of female students and 7.7% of male students (Table 2).

Changes in diet during the COVID-19 pandemic were associated with post-pandemic eating habits (Table 3). The likelihood of the increase in the consumption of both health-promoting and non-healthy foods during the pandemic increased with the frequency of consumption of the same foods after the pandemic.

The odds of decreased physical activity during the pandemic adjusted for sex and age were lower for students exercising at least four days a week than less often post-pandemic (OR 0.6; 95%CI 0.4–0.8; *p* < 0.005).

The insufficient consumption of fresh vegetables and fruits after the pandemic was evident among the study participants (Table 4). Merely half (50.3%) of the female students and approximately one-third (36.9%) of the male students reported consuming fresh vegetables on a daily basis. Similarly, about one-third of the female students (36.7%) and one-fourth of the male students (28.9%) reported consuming fruits daily. The majority of students consumed porridge several times a week or less frequently. Most students consumed meat and meat products at least several times a week. Alarmingly, nearly half of the students were found to consume confectionery, sweets, unhealthy snacks, and sweetened beverages on a daily basis or several times a week.

Females displayed a higher level of adherence to healthier eating habits compared to males. Specifically, females demonstrated a greater frequency for the consumption of fresh vegetables, fruits, and berries. However, they presented a lower frequency for the consumption of various types of meat and meat products, fermented cheese, fast food, unhealthy snacks, and sweetened beverages than their male counterparts (Table 4). One-third (32.3%) of male students and 23.2% of female students exercised post-pandemic at least four days a week (*p* < 0.001).

**Figure 1:**
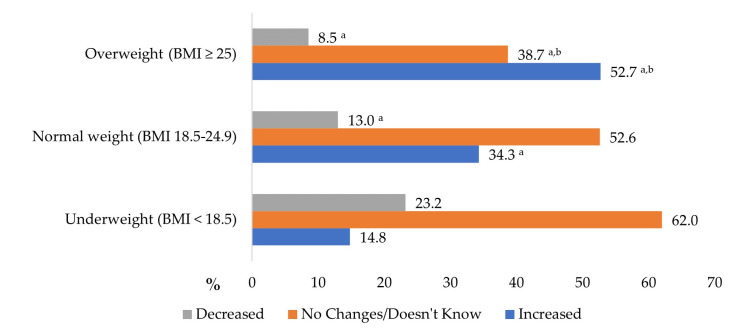
Changes in body weight during the COVID-19 pandemic compared to body weight after the pandemic (%). ^a^ *p* < 0.001 compared with underweight; ^b^ *p* < 0.001 compared with normal weight.

Most students (62.5% of males and 66.3% of females) had a normal body weight after the pandemic. Underweight was identified twice as frequently in females compared to males (11.3% and 5.2%, respectively, *p* < 0.05). Even 32.3% of males and 22.4% of females (*p* < 0.05) were overweight. The most significant increase in body weight during the COVID-19 pandemic was identified within the group of students with overweight (Figure 1). Approximately half (52.7%) of the overweight or obese students indicated an increase in their body weight during the quarantine period. Conversely, in the underweight and normal BMI groups, 14.8% and 34.3% of the students reported an increase in body weight.

Weight gain was associated with changes in nutritional habits. Among the students who reported an increased consumption of red meat during the COVID-19 pandemic, the majority (70.1%) experienced weight gain (Table 5). Moreover, an increase in the consumption of various food items, including meat products, confectionery, sweetened beverages, fast food, and snacks, as well as an increased amount of food ordered for home delivery or takeaway, was associated with the weight gain of over 60% of students. Additionally, the increased consumption of healthy foods such as fresh vegetables, fruits, berries, nuts, seeds, and cereals was also associated with weight gain; however, a smaller proportion of students gained weight while consuming these foods. On the other hand, a high proportion of students who decreased their consumption of fresh vegetables, fruits, berries, and porridge, also indicated weight gain. Decreased physical activity during the COVID-19 pandemic was strongly linked to increased body weight (Table 5).

In the multivariate logistic regression analyses, the associations between weight gain and increased intake of red meat, homemade confectionery, fast food, and snacks, as well as decreased physical activity during the COVID-19 pandemic and BMI after the pandemic, were statistically significant (Table 6). Reduced physical activity during the pandemic increased the odds of weight gain by 3.8 times, followed by the increase in the consumption of red meat, snacks, fast food, and homemade confectionery. Additionally, the odds of weight gain increased by 1.2 times for every 1 kg/m^2^ increase in BMI after the pandemic. Furthermore, significantly higher odds of weight gain were observed among female students.

**Figure 2:**
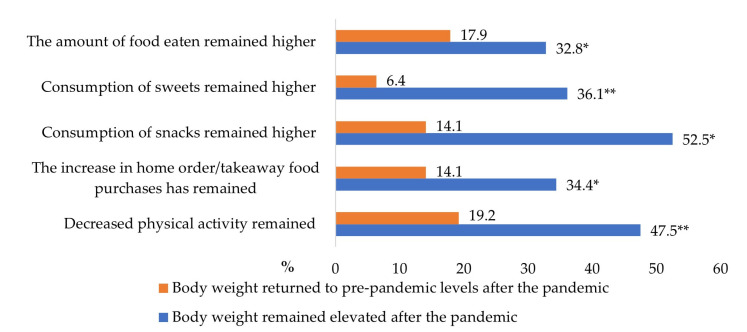
The proportion of students who maintained some lifestyle changes that occurred during the pandemic, considering changes in body weight after the pandemic (%). * *p* < 0.05, ** *p* < 0.001 compared with students, whose weight returned to pre-pandemic levels.

Even 40.9% of students who gained weight during the pandemic reported that body weight remained elevated after the pandemic. Approximately half of such students (52.5%) continued to consume snacks frequently (Figure 2). Almost a third of them answered that other unhealthy eating habits, such as the increased amount of food eaten, increased consumption of sweets, and home-ordered/takeaway food purchases, remained after the pandemic. Reduced physical activity was mentioned by 47.5% of students, whose weight remained elevated, compared to 19.2% of students, whose weight returned to pre-pandemic levels.

## 4. Discussion

Our study revealed that most students’ nutritional habits did not align with a healthy diet. Notably, there was an insufficient consumption of fresh vegetables, fruits, berries, legumes, and fish, while an excess intake of meat and meat products, confectionery, sweets, fast food, and unhealthy snacks was prevalent among students. Female students demonstrated a higher level of adherence to healthier eating habits compared to their male counterparts. Specifically, females consumed more vegetables, fruits, and berries and fewer unhealthy snacks, fast food, soft drinks, meat and meat products, and fermented cheese. The existing literature highlights that a significant number of university students fail to meet the recommended daily intake of fruits and vegetables [11,14]. They prefer processed and convenience foods, neglecting fresh and nutrient-rich options. A typical student diet is characterized by frequent skipping of breakfast and meals and the consumption of unhealthy food choices such as fast food, sugary snacks, and sugary beverages [14,15]. These dietary preferences are influenced by time constraints, busy schedules, the higher cost of healthy foods, and the comfort and accessibility of fast food [9]. Unhealthy foods are typically high in calories, saturated and trans fats, and added sugars, leading to weight gain and the development of chronic noncommunicable diseases such as cardiovascular diseases, diabetes, cancers, and others later in life. Males show a higher preference for fast food, takeaway options, and meat and meat products than females. In contrast, females tend to report healthier eating and snacking habits, often choosing fruits, vegetables, or yoghurt [9,11,13,14,15]. Females’ food choices may also be influenced by concerns about body weight and shape, which can impact their dietary decisions [15,31]. They might also be more prone to emotional eating, using food as a coping mechanism for stress, sadness, or other emotions [32]. Also, the better nutritional knowledge of female students compared to males can influence their attitudes towards healthy eating and food choices [31,33]. 

The COVID-19 pandemic had a significant impact on dietary practices worldwide [34]. The implementation of lockdowns and work from home policies modified daily routines, caused food shortages and income losses, and reduced physical activity [35]. Our study revealed notable changes in the eating habits of university students during the COVID-19 pandemic. These changes were characterized by frequent snacking; the increased consumption of fast food, homemade confectionery, and sweets; as well as a greater preference for ordering food for home delivery or takeaway. Our findings are in line with existing data from other studies. Students in various countries were found to consume insufficient amounts of healthy foods, such as cereals, dairy products, nuts, fruits, and vegetables. On the other hand, there was an increase in the consumption of confectionery, sweets, and fast food during this period [3,5,6]. Other studies also indicated a decrease in breakfast frequency, an increase in snacking between meals, and a rise in the number of daily meals [3,4]. Moreover, in Spain, some students reported a shift from morning snacking to late-night eating, with 54% of them admitting to consuming food before going to bed [7]. Similar patterns of eating were observed in Sweden, where breakfast consumption became less frequent and late-night eating increased [36].

Spending a lot of time at home, social isolation, loneliness, and stress may contribute to excessive energy intake, eating more than expected, and disruption in meal timings [37,38,39]. Irregular eating patterns, frequent snacking, and skipping breakfast led to an increase in energy intake [6,40]. During quarantine, nearly one-third of students in Germany and Italy reported increased food consumption, with pastries, bread, meat, dairy products, and fruits being the most commonly consumed items [5]. Additionally, the consumption of cheese snacks increased in Spain [7].

Our study found that one-third of students reduced physical activity during the quarantine. Following the implementation of remote learning in higher education institutions, a substantial proportion of students adopted a more sedentary lifestyle, devoting extended periods to sitting in front of electronic devices such as computers, tablets, smartphones, or television screens [17,24]. These alterations in physical activity patterns included changes in the amount of time spent on physical activities and modifications in the type and intensity of physical activity. Both male and female students were affected, with male students’ physical activity seemingly more influenced by isolation and quarantine measures [41].

During the COVID-19 pandemic, changes in students’ dietary and physical activity habits had notable implications for body weight. Our data show that more than a third of students reported body weight gain. Other authors also found that modifications in food consumption and physical activity had a negative impact on students’ BMI [26,42]. In Germany, Saudi Arabia, and Spain, approximately one-third of students experienced an increase in body weight [7,8,26]. Studies conducted in Malaysia and Indonesia found that body weight increased for every other student, while it decreased for every fifth student [43]. In Croatia, about one-fifth of students experienced weight gain, while approximately one-third experienced weight loss [17]. Some studies reported no significant changes in body weight among students [44,45,46].

In our study, more students with overweight and obesity than those with normal weight reported a weight gain. Similar findings were reported by researchers from Turkey, where 58.5% of participants experienced weight gain during the COVID-19 pandemic, with the highest increase (66.3%) observed among individuals with obesity [47]. In a Polish study, it was observed that people with higher BMI were more likely to develop unhealthy dietary habits during quarantine. Increased BMI was associated with a less frequent consumption of vegetables, fruits, and legumes during quarantine, while showing higher adherence to meat, dairy, and fast foods [48]. Another Turkish study highlighted key factors contributing to weight gain, including increased serving sizes, decreased physical activity, and an elevated number of main meals. Moreover, the consumption of white bread, packaged sweet products, and sugar was associated with weight gain [47]. A study from Belgium revealed that the increased consumption of sweet or salty snacks and reduced physical activity during the pandemic period were significant behavioral changes associated with weight gain during the COVID-19 pandemic [49]. The findings from these studies are consistent with data from our study, which identified associations between nutrition habits, physical activity, and weight changes during the COVID-19 pandemic. It is important to note that each country varies in terms of social, economic, and cultural factors. Identifying the factors that contribute to the risk of weight gain is essential for developing targeted interventions.

Our study provided evidence indicating that changes in dietary patterns during the COVID-19 pandemic were associated with post-pandemic eating behaviors. Many studies found adverse changes in dietary habits, physical activity, and body weight during the COVID-19 pandemic. However, there are a lack of data on whether these changes were transient or persisted over time and on their potential impact on the future health of the population [34]. We observed that both health-promoting and non-healthy food consumption showed a greater increase among students who continued to consume healthy food daily after the pandemic. Additionally, our data reveal that students who reported persistently elevated body weight after the pandemic were more likely to adopt unhealthy lifestyle changes—including increased snacking, consumption of sweets, larger food portions, more frequent use of home delivery or takeaway food services, and reduced levels of physical activity—during the pandemic and maintain these changes post-pandemic. This highlights the importance of paying special attention to students with overweight, encouraging and supporting them to adopt healthy lifestyle habits. Promoting a healthy diet and regular physical activity among university students is essential for developing lifelong habits that contribute to healthy body weight and overall well-being. Informational campaigns, using digital platforms and campus events, can educate students about the importance of a healthy lifestyle. Collaboration with university catering services to provide a wide range of healthy meal options is crucial. It helps students to make easier choices regarding plant-based options, whole grains, fruits, and vegetables. Accessible and well-equipped physical activity facilities at the university campus, walking, and biking paths create opportunities for different types of physical activity. Promoting healthy nutrition and physical activity among university students requires a collaborative effort from various stakeholders within the university and community. University administration plays a crucial role in supporting campus health initiatives, allocating resources, and creating policies that prioritize students’ healthy lifestyles. Student organizations can organize events and awareness campaigns that encourage their peers to adopt healthy lifestyle habits. Local healthcare professionals can become involved to provide students with evidence-based guidance related to healthy nutrition and physical activity. Further studies are needed to evaluate the long-term health effects of health promotion activities among students.

The main strength of our study lies in the assessment of nutrition, physical activity, and body weight changes, not only during the COVID-19 pandemic but also the persistence of changes post-pandemic. This approach provides a comprehensive view of how the pandemic has affected students’ lifestyles in the long term. The selection of Lithuanian applied science universities across various municipalities allowed us to represent different geographical regions of the country. Additionally, our study involved students from diverse faculties, including biomedical, social, and technological disciplines, which enhances the representativeness of the student population. 

This study has several limitations. First, data were collected using a self-administered questionnaire and non-probability sampling. Students who were willing to participate in the study might not accurately represent the whole student population. This can lead to sampling bias and limit the generalizability of findings. The reliance on self-reported data introduces the possibility of under-reporting and unintentional misreporting. Participants could not accurately recall changes in lifestyle habits during and after the COVID-19 pandemic, leading to memory bias. Additionally, respondents may provide answers that they believe are socially acceptable or desirable rather than reflecting their true behaviors. Moreover, the food frequency questionnaire has some limitations due to the inability of individuals to precisely estimate and report their long-term food intake. While some studies demonstrated that BMI calculations based on self-reported data can accurately classify a sufficient part of the population into the correct BMI categories, potential inaccuracies in reporting should still be considered [50,51]. Second, the cross-sectional study design restricts the ability to establish causal relationships between the examined variables. Finally, our study lacked information about the pre-pandemic nutrition habits, physical activity, and body weight of participating students. This information would be very useful in clarifying students’ answers about changes in lifestyle habits during and after the COVID-19 pandemic. 

## 5. Conclusions

The study revealed that most students’ nutrition habits deviated from the recommended guidelines for a healthy diet. The COVID-19 quarantine led to significant alterations in students’ eating habits, characterized by increased snacking; consumption of fast food, homemade confectionery, and sweets; and a greater reliance on home delivery or takeaway food services. Some dietary habits that emerged during the pandemic persisted in the post-pandemic period. Interestingly, this applied to both health-promoting and unhealthy habits and was linked to students’ weight indicators. Students with overweight and obesity were more susceptible to weight gain during the pandemic and maintained unhealthy changes in eating habits and physical activity after the pandemic. Thus, the promotion of a healthy diet and regular physical activity among university students is essential for improving students’ post-pandemic lifestyles to prevent health problems in the future. Additionally, future studies are needed to investigate the long-term effect of COVID-19 restrictions on students’ lifestyles and associated health issues to develop targeted strategies that ensure students’ well-being in the times ahead.

## Figures and Tables

**Table 1:** Characteristics of the study population.

Characteristics	n	%
**Gender**		
Male	325	22.7
Female	1105	77.3
**Age groups**		
18–19 years	640	44.8
20 years and older	790	55.2
**Place of study**		
Vilnius University of Applied Sciences	508	35.6
Kaunas University of Applied Sciences	579	40.5
Klaipeda State University of Applied Sciences	176	12.3
Siauliai State University of Applied Sciences	167	11.7
**Body mass index**		
<18.5 kg/m^2^	142	9.9
18.5–24.9 kg/m^2^	936	65.4
≥25 kg/m^2^	262	24.7

**Table 2:** Changes in students’ nutrition habits, physical activity and body weight during the COVID-19 pandemic (%).

Variables	Changes						*p-*Value (χ^2^ Test)
	Increased		Remained as Usual		Decreased		
	M	F	M	F	M	F	
Consumption of red meat	6.9	6.5	82.9 *	91.7	10.2 *	1.8	0.001
Consumption of porridge, flakes	14.6	11.7	77.3 *	85.2	8.1 *	3.1	0.002
Consumption of fresh vegetables	21.4 *	15.7	72.7 *	83.1	5.9 *	1.2	<0.001
Consumption of boiled, pickled vegetables	11.8 *	5.2	82.9 *	92.3	5.3 *	2.5	<0.001
Consumption of fresh fruits or berries	21.4	17.2	72.6 *	80.9	6.0 *	1.8	0.002
Consumption of nuts, seeds	12.9	11.7	79.7 *	86.5	7.4 *	1.8	0.001
Consumption of home-made confectionery	21.1 *	14.5	70.2 *	82.8	8.7 *	2.8	<0.001
Consumption of sweets	20.8	16.0	71.1 *	80.0	8.1 *	4.0	0.003
Consumption of sweetened beverages	13.2 *	7.1	69.9 *	80.3	16.9 *	12.6	0.001
Consumption of fast food	23.2 *	14.8	62.4 *	74.8	14.4	10.5	<0.001
Consumption of snacks	33.7 *	24.1	60.9 *	73.5	5.3 *	2.5	<0.001
Amount of food ordered for home delivery/takeaway	21.9	21.2	66.0	68.2	12.1	10.6	0.88
Physical activity	15.1 *	7.7	50.0 *	59.1	34.9	33.2	0.001
Body weight	31.1 *	38.7	61.2 *	46.8	7.7 *	14.5	<0.001

* *p* < 0.05 compared with females (z-test with Bonferroni correction for multiple comparisons). Abbreviations: M—males; F—females.

**Table 3:** Odds ratios for the increase in consumption of certain foods during the COVID-19 pandemic by the frequency of consumption of the same foods after the pandemic (multivariate logistic regression analysis *).

Food Products	Frequency of Consumption of the Food Product after the COVID-19 Pandemic						
	1–4 Times a Month or Less (Ref)	At Least Several Times a Week			Daily		
		OR **	95% CI	*p-*Value	OR **	95% CI	*p*-Value
Red meat	1	4.0	2.0–8.2	<0.001	7.4	3.4–16.1	<0.001
Poultry	1	4.8	2.4–9.7	<0.001	3.4	1.8–6.7	<0.001
Meat products	1	2.1	1.4–3.3	0.001	3.3	1.8–5.6	<0.001
Fish and seafood	1	3.3	2.3–4.9	<0.001	2.9	1.3–6.5	0.009
Fermented cheese	1	2.2	1.3–3.8	0.003	3.3	1.0–6.3	<0.001
Milk and milk products	1	2.0	1.1–3.4	0.02	3.1	1.8–5.4	<0.001
Porridge	1	0.3	0.2–0.4	<0.001	0.2	0.1–0.3	<0.001
Legumes	1	4.7	2.9–7.5	<0.001	9.0	4.6–17.7	<0.001
Nuts	1	4.0	2.6–6.1	<0.001	6.0	3.8–9.4	<0.001
Fresh vegetables	1	1.6	0.9–2.8	0.08	3.5	2.0–5.9	<0.001
Fresh fruits and berries	1	2.3	1.5–3.5	<0.001	4.5	3.0–6.8	<0.001
Confectionery	1	2.2	1.6–3.0	<0.001	4.3	2.8–6.7	<0.001
Sweets	1	2.8	2.0–3.9	<0.001	4.7	3.2–7.0	<0.001
Soft drinks	1	3.5	2.4–5.0	<0.001	6.7	4.3–1.5	<0.001
Energy drinks	1	7.6	5.1–11.1	<0.001	13.5	7.8–23.4	0.001
Fast food	1	3.9	3.0–5.2	<0.001	2.5	1.3–4.7	<0.001
Unhealthy snacks	1	1.5	1.1–1.9	0.004	1.5	0.9–2.6	0.09

* Separate logistic regression analysis was performed for every food item; ** OR are adjusted for sex and age. Abbreviations: OR—odds ratio, CI—confidence interval.

**Table 4:** Distribution of male and female students, according to the frequency of consumption of certain foods after the COVID-19 pandemic (%).

Food Products	Sex	Frequency of Consumption of the Food Product			*p-*Value (χ^2^ Test)
		Daily	At Least Several Times a Week	1–4 Times a Month or Less Frequently	
Red meat (pork, beef)	M	28.3 *	55.7	16.0 *	<0.001
	F	12.9	53.7	33.4	
Poultry (chicken, turkey)	M	31.9 *	54.2 *	13.9 *	<0.001
	F	17.6	63.6	18.8	
Meat products	M	15.8 *	45.8 *	38.4 *	<0.001
	F	7.9	38.7	53.4	
Fish and seafood	M	5.8 *	5.8 *	58.8 *	<0.001
	F	3.3	3.3	71.2	
Fermented cheese	M	18.3 *	46.9	34.8 *	0.01
	F	12.4	46.7	40.9	
Milk and milk products	M	33.0	44.4	22.5	0.66
	F	35.1	41.7	23.2	
Porridge	M	20.3	40.9	38.8	0.29
	F	17.8	38.7	43.5	
Legumes	M	6.8	36.4 *	56.8 *	0.008
	F	4.9	29.0	66.1	
Nuts	M	22.2	40.6 *	37.2 *	0.006
	F	19.9	33.0	47.1	
Fresh vegetables	M	36.9 *	46.8 *	16.3 *	<0.001
	F	50.3	39.4	10.3	
Fresh fruits and berries	M	28.9 *	42.5	28.6	0.02
	F	36.7	39.9	23.4	
Confectionery	M	12.3	34.5	53.2	0.15
	F	8.7	36.4	54.9	
Sweets	M	18.2	39.1	42.8	0.25
	F	14.6	42.6	42.8	
Soft drinks	M	15.8 *	15.8 *	48.3 *	<0.001
	F	9.9	9.9	63.6	
Energy drinks	M	7.1	29.1 *	63.8 *	<0.001
	F	5.3	19.1	75.6	
Fast food	M	5.8 *	33.8 *	60.3 *	<0.001
	F	2.9	21.8	75.3	
Unhealthy snacks	M	8.0 *	36.6 *	55.4 *	<0.001
	F	4.0	25.5	70.5	

* *p* < 0.05 compared with females (z-test with Bonferroni correction for multiple comparisons). Abbreviations: M—males; F—females.

**Table 5:** Proportion of students who reported weight gain caused by changes in nutrition and physical activity during the COVID-19 pandemic (%).

Variables	Changes in Health Behavior			
	Increased	Remained as Usual	Decreased	*p*-Value (χ^2^ Test)
Consumption of red meat	70.1	34.2 *	37.8 *	<0.001
Consumption of meat products	63.4	34.2 *	38.4 *	<0.001
Consumption of porridge, flakes	48.7	33.3 *	54.5	<0.001
Consumption of fresh vegetables	55.4	30.9 *	55.1	<0.001
Consumption of fresh fruits or berries	53.8	31.4 *	51.4	<0.001
Consumption of nuts, seeds	57.2	32.5 *	53.4	<0.001
Consumption of purchased confectionery	63.5	31.6 *	36.1 *	<0.001
Consumption of home-made confectionery	58.6	31.2 *	36.2 *	<0.001
Consumption of sweets	59.9	30.8 *	36.3 *	<0.001
Consumption of sweetened beverages	64.7	30.9 *	43.5 *	<0.001
Consumption of fast food	61.2	27.7 *	43.5 *	<0.001
Consumption of snacks	60.4	26.2 *	25.4 *	<0.001
Amount of food ordered for home delivery/takeaway	65.3	20.5 *	51.0	<0.001
Physical activity	35.4	20.8 *	61.9 *	<0.001

* *p* < 0.05 compared with increased health behavior (z-test with Bonferroni correction for multiple comparisons).

**Table 6:** Odds ratios (OR) for weight gain caused by sex, changes in some health behaviors during the COVID-19 pandemic and BMI after pandemic (multivariate logistic regression analysis).

Variables	OR	95% CI	*p*-Value
Females vs. males	1.5	1.1–2.1	0.007
Increased consumption of red meat *	2.6	1.6–4.4	<0.001
Increased consumption of homemade confectionery *	1.5	1.1–2.1	0.01
Increased consumption of fast food *	1.7	1.2–2.4	0.003
Increased consumption of snacks *	1.9	1.3–2.6	<0.001
Decreased physical activity **	3.8	2.9–5.0	<0.001
BMI after COVID 19 pandemic #	1.2	1.1–1.2	<0.001

* Reference variable—food consumption decreased or remained the same. ** Reference variable—physical activity increased or remained the same. # OR were calculated for a one-unit increase in the BMI. Abbreviations: OR—odds ratio, CI—confidence interval.

## Data Availability

The data presented in this study are available on request from the corresponding author. The data are not publicly available due to ethical issues.
